# Complete Genome Sequence of a Novel *Myoviridae* Phage, SfΦ01, Infecting Shigella spp.

**DOI:** 10.1128/MRA.00349-19

**Published:** 2019-06-06

**Authors:** Masaaki Kitajima, Satoshi Ishii, Tatsuma Takagi, Satoshi Okabe

**Affiliations:** a Division of Environmental Engineering, Faculty of Engineering, Hokkaido University, Sapporo, Hokkaido, Japan; b BioTechnology Institute, University of Minnesota, St. Paul, Minnesota, USA; c Department of Soil, Water and Climate, University of Minnesota, St. Paul, Minnesota, USA; Loyola University Chicago

## Abstract

The Shigella bacterium is one of the most significant causes of waterborne and foodborne bacterial dysentery. A lytic bacteriophage infecting Shigella flexneri was isolated from wastewater in Japan. We report here the complete genome sequence of this bacteriophage, revealing that it belongs to the *Myoviridae* family and possesses linear genomic DNA.

## Abstract (precis)

The Shigella bacterium is one of the most significant causes of waterborne and foodborne bacterial dysentery. A lytic bacteriophage infecting Shigella flexneri was isolated from wastewater in Japan.

## ANNOUNCEMENT

The Shigella bacterium is one of the most significant causes of waterborne and foodborne bacterial dysentery in the world (1). Among the four species of the genus *Shigella*, S. flexneri is most commonly associated with shigellosis outbreaks in the developing world (2). Bacteriophages have been proposed as a means for treating bacterial disease (bacteriophage-based therapy) (3–5), detecting and typing bacteria (6), and decontaminating surfaces and water (7, 8). We report here the complete genome sequence of a novel bacteriophage, SfΦ01, that infects S. flexneri and was isolated from wastewater in Japan.

**FIG 1:**
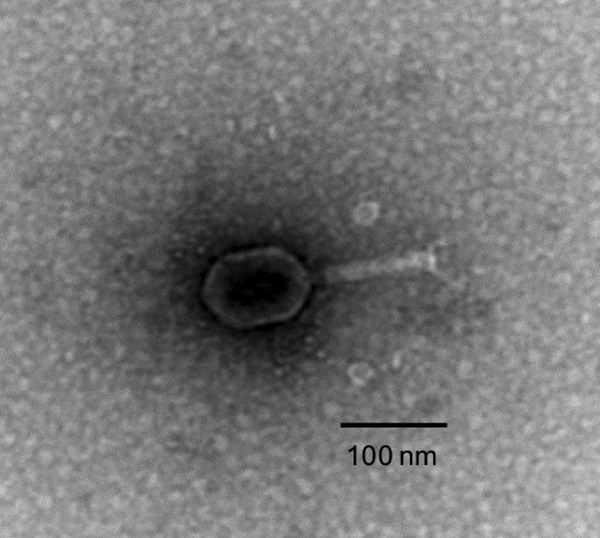
Transmission electron microscope image of bacteriophage SfΦ01 taken at ×100,000 magnification.

Bacteriophage SfΦ01 was isolated from municipal wastewater by serial plaque purification using S. flexneri (strain identifier, RIMD 3102037) as a host bacterium grown in R2A agar and incubated overnight at 37°C. Spot tests using other bacterial strains (all grown in R2A agar at 37°C) demonstrated that bacteriophage SfΦ01 is capable of infecting Shigella sonnei (RIMD 3104005) and Escherichia coli O1:K1:H7 (JCM1649) as well. Replication of bacteriophage SfΦ01 in these bacterial strains was confirmed by an increase in bacteriophage SfΦ01 genome copy numbers after infection (data not shown). However, this bacteriophage did not infect other types of E. coli, such as the E. coli K12 (MG1655), O26:H11 (RIMD 05091992), O111, and O157:H7 Sakai (RIM0509952) strains. Electron micrographs of bacteriophage particles showed that SfΦ01 had an icosahedral head with a contractile tail (Fig. 1), which morphologically resembled bacteriophages belonging to the family *Myoviridae* (in the order *Caudovirales*) (9).

For genomic DNA extraction, bacteriophage SfΦ01 was inoculated to S. flexneri grown in R2A medium and incubated overnight at 37°C. Bacterial cells were removed by centrifugation and filtration with a 0.45-μm-pore-size filter. Bacteriophage particles were concentrated using the Centricon Plus-70 filter (Merck Millipore), and 160 μl of bacteriophage concentrate was mixed with 20 μl of DNase I (Promega) to digest free DNA. Bacteriophage genomic DNA was extracted from the resultant sample using the PowerBiofilm DNA isolation kit (Mo Bio Laboratories). Sequencing libraries were prepared using the TruSeq PCR-free library prep kit (Illumina) with an insert fragment size of ca. 350 bp and paired-end sequenced by using the MiSeq platform (Illumina) with v2 chemistry (250 cycles). The sequencing reads (541,594 reads each for forward and reverse sequencing reactions) were assembled *de novo* by using the SPAdes v. 3.12 program (10). The genome assembly depth (coverage) was 1,618. Genes were predicted by using PHANOTATE (11) and translated and annotated by using in-house perl scripts and a BLASTP algorithm against the nonredundant (nr) database. Default parameters were used for all software tools.

The sequencing results revealed that the genome of bacteriophage SfΦ01 is double-stranded linear DNA with a size of 168,000 bp and a G+C content of 35.29% and containing 288 protein-coding sequences (CDSs). A BLASTn search of the complete genome of SfΦ01 showed the highest identity of 95.58% with *Shigella* phage Sf21 (GenBank accession number MF327007), which belongs to the family *Myoviridae* and possesses a linear genome. The present study provides the complete genome sequence information of a novel bacteriophage, SfΦ01, infecting *Shigella* spp.

### Data availability.

The complete genome sequence of bacteriophage SfΦ01 has been deposited in the NCBI database under the GenBank accession number LC465543. Raw data corresponding to the bacteriophage SfΦ01 genome were deposited in the DDBJ DRA database under the SRA accession number DRR175055.
